# Gentrification and Displacement in the San Francisco Bay Area: A Comparison of Measurement Approaches

**DOI:** 10.3390/ijerph16122246

**Published:** 2019-06-25

**Authors:** Mahasin S. Mujahid, Elizabeth Kelley Sohn, Jacob Izenberg, Xing Gao, Melody E. Tulier, Matthew M. Lee, Irene H. Yen

**Affiliations:** 1 Division of Epidemiology, Berkeley School of Public Health, University of California, 2121 Berkeley Way West, Berkeley, CA 94720-7360, USA; xing_gao@berkeley.edu (X.G.); matt.lee94@berkeley.edu (M.M.L.); 2 Kaiser Permanente, Oakland, CA 94611, USA; eakelley2@gmail.com; 3 Department of Psychiatry, San Francisco School of Medicine, University of California, 982 Mission St., San Francisco, CA 94103, USA; jacob.izenberg@ucsf.edu; 4 Center for Interdisciplinary Research on AIDS, Yale University, New Haven, CT 06510, USA; melody.tulier@yale.edu; 5 University of California, Merced, Public Health, 5200 N. Lake Road Merced, CA 95343, USA; iyen@ucmerced.edu

**Keywords:** gentrification, neighborhoods, health and health disparities

## Abstract

Gentrification may play an important role in influencing health outcomes, but few studies have examined these associations. One major barrier to producing empirical evidence to establish this link is that there is little consensus on how to measure gentrification. To address this barrier, we compared three gentrification classification methodologies in relation to their ability to identify neighborhood gentrification in nine San Francisco Bay Area counties: the Freeman method, the Landis method, and the Urban Displacement Project (UDP) Regional Early Warning System. In the 1580 census tracts, 43% of the population had a bachelor’s degree or higher. The average median household income was $79,671 in 2013. A comparison of gentrification methodologies revealed that the Landis and Freeman methodologies characterized the vast majority of census tracts as stable, and only 5.2% and 6.1% of tracts as gentrifying. UDP characterized 46.7% of tracts at risk, undergoing, or experiencing advanced stages of gentrification and displacement. There was substantial variation in the geographic location of tracts identified as gentrifying across methods. Given the variation in characterizations of gentrification across measures, studies evaluating associations between gentrification and health should consider using multiple measures of gentrification to examine the robustness of the study findings across measures.

## 1. Introduction

It has been well established that place impacts health [1,2]. An extensive body of research has documented that neighborhood physical and social environments are associated with a myriad of health outcomes over the lifecourse (e.g., pre-term birth, physical and mental health in childhood and adulthood, mortality) [1,3,4,5,6] and are important root causes of racial/ethnic health inequalities [7,8,9]. As a result, efforts to improve neighborhood environments are being leveraged in order to achieve health equity [10,11]. Their success is predicated on understanding the structural processes that accompany neighborhood revitalization and change initiatives. Gentrification is one such process that is highly controversial and hotly debated, given its potential to be both beneficial and harmful to community residents [12,13,14,15]. 

Gentrification is a process through which lower-income neighborhoods experience capital investments and an influx of wealthier residents [16,17,18]. New investments, business developments, and increases in the tax base all have the potential to reinvigorate neighborhoods that have experienced historical disinvestment and to provide significant capital gains. However, these improvements may come at the expense of long-time residents and businesses, who are forced to move as their neighborhoods become unaffordable [15,19]. The extent of displacement is unknown but nonetheless has the potential to alter the community fabric, sever social support networks, and reduce social capital for the most vulnerable residents, including the poor, elderly, people living with disabilities, and racial/ethnic minorities [15,19,20,21]. 

**Figure 1:**
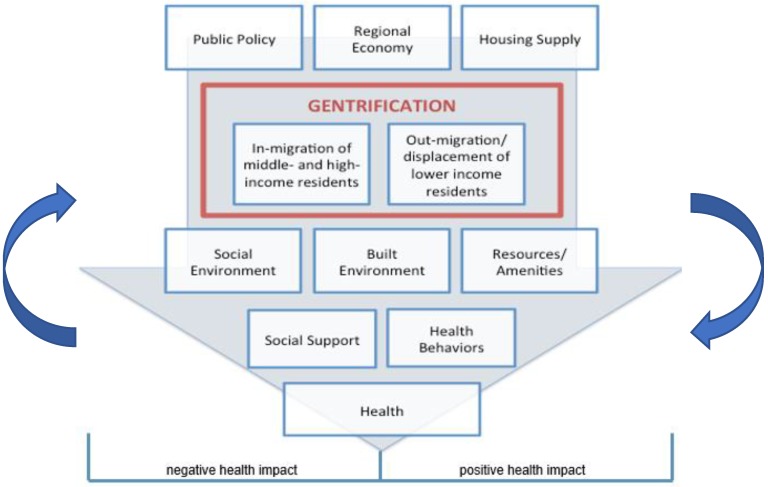
The conceptual framework of the potential health impacts of gentrification.

The ways in which gentrification and displacement may impact health are understudied with important implications. Gentrification can affect health through multiple pathways by altering the built environment and health-promoting resources, as well as by affecting the community social environment (Figure 1) [19]. For instance, gentrification can affect the placement and upkeep of community resources vital to health, such as neighborhood parks and playgrounds that encourage physical activity or grocery stores that supply healthy food. These health-promoting resources have been shown to have beneficial impacts on health [22,23]. Gentrification can also affect community social cohesion when long-time residents, integral members of social networks of trust and support, move out as a result of involuntary displacement. Gentrification-driven displacement of vulnerable residents, who often have the worst health profiles, may contribute to widening health disparities [19]. This is complicated further by the fact that government-led investment initiatives may themselves precede and/or promote widespread gentrification, leading to further changes in the built and social environments. Earlier investments may indeed be beneficial to the neighborhood’s longtime residents, while the effects of subsequent changes may be more uneven, helping some populations while harming others who are marginalized or displaced by the altered landscape.

In order to develop a better understanding of gentrification, it is first necessary to conceptualize and measure it; currently, there is little consensus regarding the best methodology. Some studies have measured gentrification using aggregate census data that document changes in median household income [24] or changes in occupation composition of the census tract such as the proportion of professionals [25]. Others have used more complex methodologies that combine census data and home sales data to focus on demographic change as well as changes in amenities and transit. These methods take into account location, accessibility, and the role of displacement [15,26,27]. Which of these measures best captures the concept of gentrification and its association with health outcomes remains understudied [28].

Thus, the overall goal of this study was to compare and contrast three gentrification classification methodologies in relation to their ability to identify neighborhood gentrification in nine San Francisco Bay Area counties between 2000 and 2013. The San Francisco Bay Area is one metropolitan region in the United States that is experiencing these trends acutely. The cost of living in the Bay Area has risen dramatically. Gentrification, fueled by the influx of high-salaried technology employees combined with a shortfall of housing stock, has become a contentious issue in historically lower-income neighborhoods [29]. Developing a better understanding of the implications of gentrification and displacement for health in the San Francisco Bay Area may provide insight for other cities experiencing similar circumstances. By comparing how different methodologies characterize changes occurring at the neighborhood level, we can begin to discern how the decisions we make in conceptualizing and measuring gentrification might affect health and health inequalities.

## 2. Materials and Methods

### 2.1. Setting

We examined census tracts in the nine Bay Area counties, which include Alameda, Contra Costa, Marin, Napa, Santa Clara, San Francisco, San Mateo, Solano, and Sonoma counties. This region is geographically diverse and contains urban, suburban, and rural areas, with a total population of 7,257,501 people in 2013. This region is also one of the most racially and ethnically diverse regions in the country [30]. 

Over the past several decades, there have been great demographic and economic shifts in the Bay Area. In particular, there has been significant population growth, largely fueled by an influx of people of color moving to the region [28]. Asian Americans and Hispanics/Latinos have contributed to much of this growth [28]. In 1980, no county in the Bay Area had <50% non-Hispanic White residents, whereas currently, all counties except for Marin county have ≤50% non-Hispanic White residents [30]. The Bay Area has also undergone significant economic growth over the past several decades, accompanied by a sharp increase in income inequality. Since 1979, the highest paid workers in the region have experienced significant growth in wages, while the wages of the lowest paid workers have declined [28].

### 2.2. Measuring Gentrification

We employed three measures of neighborhood gentrification. The Freeman and Landis measures are well-established measures of gentrification and neighborhood change. We also included a more contemporary measure from the Urban Displacement Project (UDP), which provides a more nuanced view of the stages of gentrification. Details on the construction of the three measures can be found in Table S1. We defined neighborhoods as census tracts given robust evidence that tract-level geographic boundaries have been shown to be predictive of health. Below we describe the unique features of each measure as well as their strengths and limitations.

#### 2.2.1. Freeman Method

The Freeman Method is considered the gold standard of measuring neighborhood gentrification [15]. Census tracts were defined as gentrifiable if at the beginning of a defined period (year 2000, based on the US Census), it met the specified inclusion criteria: (1) the tract was at or below the median income for its respective metropolitan area, (2) the percentage of housing stock within the tract built in the prior twenty years was at or below the median for all census tracts in the metropolitan area, and (3) at least 50% of the census blocks within the tract were defined as urban.

We made two slight modifications to the Freeman method. First, we chose to use a relatively large area, the San Jose—San Francisco—Oakland Combined Statistical Area (CSA) as the metropolitan area to simplify the analysis. Whereas the borders of individual metropolitan areas have changed substantially between 2000 and 2013, the CSA boundaries have remained stable, simplifying the measurement of variables over time. This CSA also closely approximated the nine-county Bay Area. Second, we used a 50% urban cutoff for inclusion, rather than the “central city” designation employed by Freeman. This decision was rooted in our desire to include as many of the nine-county census tracts as reasonably possible and to use publicly available census data, rather than relying on other data sources, in order to maximize the generalizability of this approach. 

We further categorized census tracts that met eligibility criteria for being gentrifiable as having either gentrified or not during the 2010–2013 period, a timeframe chosen to match the period analyzed by the UDP. Tracts were considered as gentrifying if they (1) saw an increase in median home sale price over the period in question and (2) saw an increase in educational attainment (those at least 25 years old possessing a bachelor’s degree or higher) exceeding that of the metropolitan area as a whole. The benefit of the Freeman measure, which serves as the basis for many more contemporary measures of gentrification, is that it is rooted in a strong conceptual framework and critical historical analysis. It can also be readily generalized to other geographic areas. However, an important limitation is its inability to characterize gentrification in non-urban areas, which limits its usefulness in characterizing such processes in more suburban and rural areas. The Freeman measure also does not capture stages of gentrification.

#### 2.2.2. Landis Method

The Landis 3-D methodology [24] is considered a general measure of neighborhood change based on the median household income of the census tract. Using data from the 1990 and 2000 U.S. Census and 2013 American Community Survey, we characterized census tracts as gentrifying when their 1990 median household income was in the lowest four deciles and their 2013 median household income had improved by two or more deciles. Neighborhoods were classified as declining if their 1990 median household incomes were in the top four deciles and their 2013 median household incomes had declined by two or more deciles. If the tracts did not fall into the previous two categories of gentrifying or declining, we classified them as stable. The Landis method’s strength is that it is a clearly defined measure of neighborhood change that only relies on minimal information: median household income. However, limitations include concerns about whether it is actually capturing the process of gentrification. Additionally, it measures the relative change in deciles rather than absolute change, which may mask patterns that occur relatively uniformly across a particular region. Finally, it does not provide information on areas that are at risk of gentrification or in advanced stages of gentrification. 

#### 2.2.3. Urban Displacement Project

To examine neighborhood change using the Urban Displacement Project, we accessed the Regional Early Warning System dataset available at http://www.urbandisplacement.org/sites/default/files/images/cci_rews_data_2015-08-21.xlsx. This dataset included a variety of variables related to gentrification and displacement for census tracts in the nine San Francisco Bay Area counties derived from a variety of sources including the census, American Community Survey, Nets, and Dataquick. Additional details regarding this data and the Regional Early Warning System methodology are explained elsewhere [27]. Briefly, using a mix of Census and home sales data, this methodology characterizes census tracts into eight typologies. Low-income tracts, defined as those with >39% low-income households, were categorized as (a) not losing low-income households or at the very early stages of gentrification, (b) at risk of displacement, (c) undergoing displacement, or (d) advanced stages of gentrification. Moderate-to-high income tracts, defined as <39% low-income households, were categorized as: (a) not losing low-income households or very early stages of gentrification, (b) at risk of displacement, (c) undergoing displacement, or (d) advanced exclusion [25]. A small subset of tracts (*N* = 11) was not classified due to designation as being a college-town based on criteria specified by UDP. A key strength of this methodology is that it takes into account a variety of factors that have been identified as part of the processes of gentrification and displacement such as the nature of a neighborhood’s existing housing stock (% of prewar buildings) and its proximity to jobs and transit [27]. In addition, this methodology takes into account neighborhood changes that may provide early warning signs of displacement, including neighborhood-level loss of market rate affordable housing units, housing price appreciation, and market rate development [27]. However, the reliability of this methodology relies somewhat on the researchers’ nuanced and deep familiarity with the San Francisco Bay Area, as well as access to multiple sources of data that are not publicly available. This limits the replicability of the methodology for use in cities across the country. 

### 2.3. Data Analysis

To compare and contrast the three measures of gentrification, we first examined the sociodemographic characteristic of tracts (e.g., median household income, racial composition) overall and by each of the gentrification measures, using data from the 2013 American Community Survey 5-year estimates. Next we used cross-tabulation to compare each of the three measures of gentrification. Finally, we used ArcGIS to create spatial maps for the gentrification typologies for all three measures.

## 3. Results

There were 1580 tracts corresponding to 7,257,501 people in the nine counties in the Bay Area in 2013. Across these tracts, the median household income was $79,671, the mean percentage of the population living below poverty across tracts was 11.5%, and the mean racial composition was 43.4% white, 6.7% black, 22.7% Asian, 22.6% Hispanic, and 4.6% other or multi-race.

Table 1 shows the demographics of the neighborhoods by the Freeman method of gentrification. Based on this methodology, 6.1% of neighborhoods were gentrifying, 5.8% were not gentrifying, and 88.0% were ineligible. Of the 1391 neighborhoods that were excluded, 1270 were excluded because they were medium or high income tracts, one tract was excluded because it was not in an urban area, and another 120 tracts were excluded because the proportion of housing built in the prior 20 years was greater than the median across all neighborhoods. 

Tracts categorized as not gentrifying had an 11% decline in median household income between 2000 and 2013, while median household income in gentrifying tracts increased by 4%. Gentrifying tracts had a larger increase in the proportion of college-educated adults between 2000 and 2013, compared to tracts categorized as not gentrifying. Tracts categorized as not gentrifying had larger increases in the proportion of non-white residents and the proportion of renter households between 2000 and 2013, compared to gentrifying tracts. 

Descriptive statistics for Bay Area neighborhoods categorized according to the Landis methodology are presented in Table 2. Based on this methodology, 91.2% of neighborhoods were categorized as stable, 5.2% of the neighborhoods were categorized as gentrifying, and 3.5% of the neighborhood were categorized as declining. Declining neighborhoods had the greatest decrease in median household income, while gentrifying neighborhoods had the greatest increase in median household income between 2000 and 2013. Stable and gentrifying neighborhoods had relatively similar proportions of non-white populations in 2013, while declining neighborhoods had a higher proportion of non-white residents. Declining, stable, and gentrifying neighborhoods all experienced growth in the non-white population between 2000 and 2013, with the largest percent change occurring in declining census tracts. Stable and gentrifying neighborhoods experienced growth in the proportion of college-educated adults, with the largest growth in gentrifying census tracts. Declining census tracts had the largest growth in the proportion of renter households between 2000 and 2013.

Descriptive statistics for the Urban Displacement Project neighborhood typologies are presented below in Table 3. The distribution of neighborhoods across categories are as follows: 20.8% of all census tracts were low-income tracts that were not losing low-income households, 18.4% were at risk of gentrification or displacement, 4% were undergoing displacement, and 9.4% were experiencing advanced gentrification. 30.1% of all census tracts were middle-to-high income tracts that were not losing low-income households, 8.4% were at risk of displacement, 6.5% were undergoing displacement, and 1.8% were experienced advanced exclusion. Thus, 46.7% of the Bay Area’s neighborhoods were characterized as either being at risk, undergoing, or in the advanced stages of gentrification and displacement. 

All neighborhood typologies experienced a growth in the proportion of non-white population and the proportion of adults with college education between 2000 and 2013 (Table 3). Three neighborhood typologies experienced a decline in median household income between 2000 and 2013: low-income tracts that are not losing low-income households or are at the very early stages of displacement, low-income tracts at risk of gentrification or displacement, and medium/high income tracts that are not losing low-income households or are at very early stages of displacement. Medium/high income neighborhoods that were categorized as undergoing displacement or advanced exclusion had the greatest loss in the proportion of low-income households between 2000 and 2013.

The cross-tabulation of neighborhood categorization according to the Freeman, Landis, and UDP methodology are presented in Table 4. Among neighborhoods that were gentrifying according to Freeman (*N* = 97), 44.3% of them were also classified as at risk of gentrification or displacement, and 33.0% of them were classified as experienced advanced gentrification by UDP. However, among those neighborhoods classified as gentrifying according to Landis (*N* = 82), 42% were classified as low-income neighborhoods that were experiencing advanced gentrification by UDP and 32.1% were classified as being medium-to-high income neighborhoods that were at risk of or undergoing displacement. Thus, the majority of gentrifying neighborhoods classified by Landis and Freeman were also classified as gentrifying by UDP. It is important to note that although a similar proportion of neighborhoods were classified as gentrifying by both the Freeman and Landis measures (6.1% for Freeman and 5.6% for Landis), the neighborhoods themselves were not the same. Among neighborhoods that were classified as gentrifying based on Freeman (*N* = 97), only 13.4% of them were also classified as gentrifying based on Landis and the remaining 86.6% were classified as stable (data not shown).

Both the Freeman and Landis methodologies did not identify some neighborhoods that were categorized as at risk of gentrifying by the UDP methodology as gentrifying (Table 4). Among those neighborhoods classified as not gentrifying (*N* = 92) and excluded (*N* = 1391) according to Freeman, 53.3% and 14.2% of them respectively were classified as low-income neighborhoods at risk of gentrification or displacement by UDP. Among those neighborhoods classified as stable (*N* = 1442) by the Landis methodology, 20% of them were classified as low-income tracts at risk of gentrification or displacement by UDP. 

**Figure 2:**
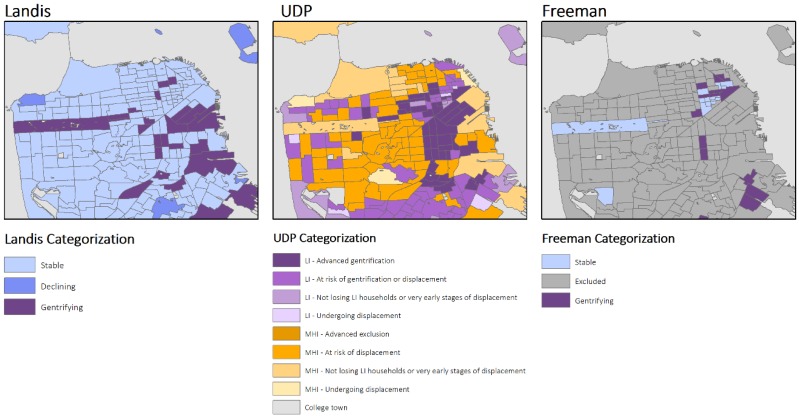
Spatial representation of Gentrification in San Francisco using the three different measures, Bay Area Census Tracts, 2000–2013.

**Figure 3:**
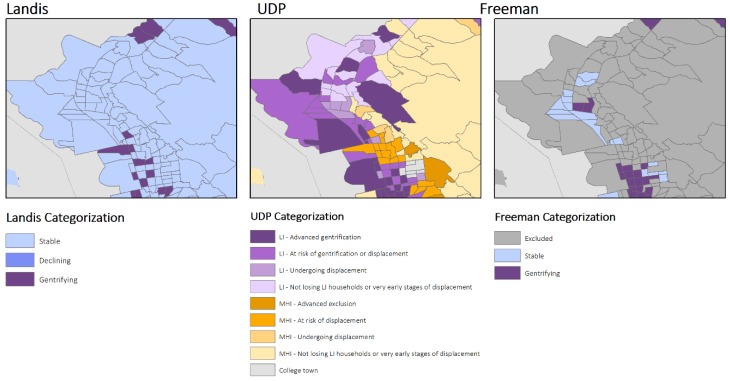
Spatial Representation of Gentrification in Richmond, California using the three different measures, Bay Area Census Tracts, 2000–2013.

**Figure 4:**
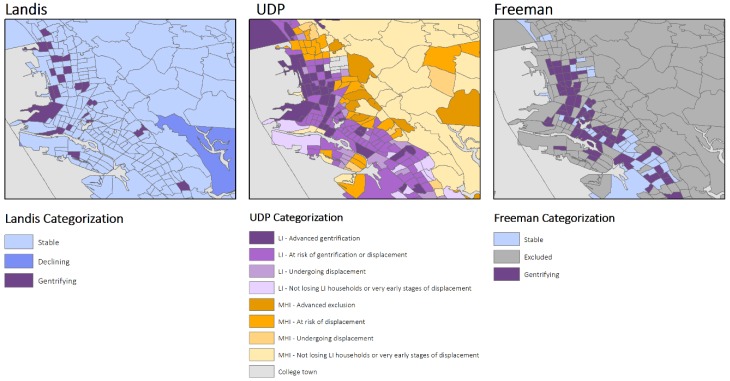
Spatial representation of Gentrification in Oakland, California using the three different measures, Bay Area Census Tracts, 2000–2013.

Variations in measuring gentrification resulted in both complementary and contradictory visual representations of the process of gentrification in the Bay Area for three cities: San Francisco, Richmond, and Oakland CA (Figure 2, Figure 3 and Figure 4). Visually, the UDP approach indicated a greater number of neighborhoods experiencing gentrification throughout the region. In turn, generally the neighborhoods that are classified by Freeman and Landis as gentrifying are also classified as such by UDP. However, in areas experiencing recent re-zoning and growth, the methodologies provide distinct results. For example, within the West Berkeley, Emeryville, and West Oakland area, which are located on the western portion of the map of Oakland (Figure 4), the UDP indicates advanced gentrification of a large portion of the area. This, in fact, reflects the re-zoning efforts catalyzing residential, manufacturing, retail, and office development. Yet, this growth is not reflected in maps of gentrification based on Landis’ measure, given its focus on changes in income of the population over time. As another illustration of the UDP’s ability to identify changing urban census tracts, a comparison of the methodologies for the area of San Francisco again shows diversion (Figure 2). Specifically, the eastern portion of San Francisco, which includes the Mission District and South of Market (SoMA), has undergone a transformation in expanding housing and light industrial use, reflected in the relatively large area properly identified by UDP as experiencing advanced gentrification. In contrast, Freeman’s methodology excludes several neighborhoods in the Mission District and SoMa, masking the process of gentrification.

## 4. Discussion

Given the increased interest in understanding the health impacts of gentrification and displacement, we sought to compare and contrast three classification methodologies: the Freeman, Landis, and Urban Displacement Project measures in the San Francisco Bay Area. Several noteworthy results emerged from these investigations. First, despite the great economic and demographic shifts that occurred in the Bay Area between 2000 and 2013, only a small proportion of neighborhoods were characterized as gentrifying based on the Freeman and Landis methodologies (6.1% and 5.2% respectively). Alternatively, the UDP characterized 19.9% of the Bay Area’s neighborhoods as undergoing or in the advanced stages of gentrification and displacement. Second, we found very little overlap in the geographic location of these neighborhoods across all three measures. The most overlap existed between UDP and either Freeman or Landis. Finally, the UDP method provided an alarming estimate that an additional 26.8% of tracts are at risk of gentrification or displacement. Thus, based on UDP, 46.7% of neighborhoods in the Bay Area were either at risk of, undergoing, or experiencing advanced stages of gentrification and displacement.

To our knowledge, this is the first study to compare and contrast 3 measures of gentrification and one of the few studies conducted outside of New York City. A study by Barton compared two census-based (Bostic and Martin, Freeman) and one qualitative measure of gentrification from 1980–2009 in New York City. Results suggested that the number of gentrified areas and the geographic distributions of those areas varied greatly between the two census-based approaches and between the census-based and qualitative approaches [28]. Another study by Freeman et al. also found substantial differences in the classification of gentrified neighborhoods comparing two census-based methodologies (Freeman, Hammel, and Wyly) [15,31]. However, despite these differences, both measures produced similar associations with study outcomes [32]. 

Notably, although Landis and Freeman’s measures provided a similar prevalence of gentrification in the San Francisco Bay Area, the location of those neighborhoods varied substantially. This is most likely a function of the Landis method not being a measure of gentrification but simply any neighborhood change, albeit a high threshold of change (i.e., a 4 decile increase). Moreover, both Freeman and Landis classification methods excluded neighborhoods that were considered at risk of gentrification based on the UDP classification. It is important to note that a unique feature of the UDP is the ability to provide early markers of gentrification and flag areas as at risk of gentrification. However, the benefit from this feature of “early warning” is offset by the challenges in replicating this measure for other cities and areas as it requires a great deal of local knowledge and non-publicly available data sources. 

While there is no widely accepted measure of gentrification, Freeman has been frequently used. A recent report in 2015 documenting the prevalence of gentrification in the 50 largest cities in the United States used the Freeman measure of gentrification. Three of the Bay Area cities were included in this report, with a prevalence of 1.5% in San Francisco, 3.6% in San Jose, and 21.2% in Oakland [33]. Moreover, studies have begun to document associations between gentrification and health behaviors and outcomes using the Freeman classification of gentrification (or measured closely based on Freeman). As an example, a study by Izenberg et al., 2016, which analyzed California Health Interview Study data reported a higher odds of fair and poor self-rated health among African Americans that lived in gentrifying neighborhoods compared to those living in non-gentrifying neighborhoods but no associations for all other racial/ethnic groups [33,34,35] Similarly, another study by Gibbons et. al., 2016, used data from a household survey in Philadelphia, Pennsylvania to document that in the general population, there was a positive association between gentrification and self-reported health. However, among black participants, gentrification was associated with worse self-rated health [36]. Given the controversial nature of this topic, it is important to ensure that results are robust to measures of gentrification. 

A few limitations of our study warrant comment. First, although we compared three measures of gentrification, the measures we selected may not be the most relevant measures in the contemporary literature and some might argue are not truly capturing gentrification (e.g., the Landis measure). As an example, Hwang et. al., 2015 developed a measure of gentrification that is theoretically grounded, technologically advanced through the use of google street view, and able to capture the stages of gentrification [37]. Thus, future work should consider comparisons across a broader range of old and new measures of gentrification. Second, we defined neighborhoods as census tracts. Although these geographic boundaries have been useful in documenting associations between neighborhood environments and health, it is unclear if these are meaningful boundaries for capturing the process of gentrification and how it unfolds over time. Finally, we investigated gentrification in the San Francisco Bay Area which provided racial/ethnic and geographic diversity. However, the Bay Area also includes many suburban and rural areas, which were classified as ineligible or not gentrifiable based on the Freeman criteria. Investigations of whether or not processes of gentrification can occur in suburban and rural communities is an important area for future work. 

## 5. Conclusions

In this paper, we were able to document vastly different estimates of gentrification across measures. As interests in the health implications of gentrification continue to rise, future studies should continue to investigate associations between gentrification and displacement on a broad range of health outcomes and how associations vary by indicators of vulnerability. Critical to this work will be a comparison of associations using several measures of gentrification to see if findings are robust to these approaches. Developing a better understanding of the implications of gentrification for health, and how these might be unequally distributed across the population, is necessary in order to inform decisions made by policymakers, urban planners, developers, and others to mitigate any ill effects associated with gentrification.

## Figures and Tables

**Table 1:** The demographics of Freeman Gentrification Typologies, the San Francisco Bay Area 2000–2013.

	Not Gentrifying	Gentrifying	Excluded
	(*N* = 92)	(*N* = 97)	(*N* = 1391)
Population (#, 2013)	384,152	363,446	6,509,903
% of Total Population Living in Category (2013)	5.3%	5.0%	89.7%
Median Income (2013)	$39,078	$48,774	$90,635
Change in median household income (%, 2000–2013)	−11%	4%	−6%
Low-income households (%, 2013)	67%	59%	38%
Change in proportion of low-income households (%, 2000–2013)	3%	−9%	3%
Non-white population (%, 2013)	75%	67%	55%
Change in non-white population (%, 2000–2013)	7%	−4%	16%
Adults (25+) with college degree (%, 2013)	22%	37%	44%
Change in college-educated adult population (%, 2000–2013)	4%	32%	12%
Renter households (%, 2013)	69%	69%	40%
Change in renter households (%, 2000–2013)	6%	−1%	3%

**Table 2:** The demographics of the Landis (3-D) Gentrification Typologies, the San Francisco Bay Area 2000–2013.

	Declining (*N* = 56)	Stable (*N* = 1442)	Gentrifying (*N* = 82)
Population (#, 2013)	234,666	6,693,536	329,299
% of Total Population Living in Category (2013)	3%	92%	5%
Median Income (2013)	$72,918	$85,340	$88,486
Change in median household income (%, 2000–2013)	−35%	−7%	42%
Low-income households (%, 2013)	44%	41%	36%
Change in proportion of low-income households (%, 2000–2013)	53%	8%	−25%
Non-white population (%, 2013)	64%	56%	53%
Change in non-white population (%, 2000–2013)	31%	23%	10%
Adults (25+) with college degree (%, 2013)	35%	43%	50%
Change in college-educated adult population (%, 2000–2013)	2%	22%	55%
Renter households (%, 2013)	38%	43%	51%
Change in renter households (%, 2000–2013)	60%	14%	−5%

**Table 3:** The demographics of the Urban Displacement Neighborhood Typologies, the San Francisco Bay Area 2000–2013.

	Low Income				Medium/High Income			
	Not Losing Low-Income Households or Very Early Stages of Displacement (*N* = 329)	At Risk (*N* = 290)	Undergoing (*N* = 63)	Advanced (*N* = 149)	Not Losing Low-Income Households or Very Early Stages of Displacement (*N* = 476)	At Risk (*N* = 132)	Undergoing (*N* = 102)	Advanced (*N* = 28)
Population (#, 2013)	1,528,330	1,324,550	279,821	660,038	2,259,243	570,934	465,156	119,329
% of Total Population Living in Category (2013)	21%	18%	4%	9%	31%	8%	6%	2%
Median Income (2013)	$60,727	$55,15	$66,208	$71,264	$112,466	$102,540	$110,760	$175,259
Change in median household income (%, 2000–2013)	−19%	−13%	3%	10%	−4%	7%	8%	4%
Low-income households (%, 2013)	54%	56%	50%	47%	27%	30%	27%	13%
Change in proportion of low-income households (%, 2000–2013)	20%	11%	−8%	−6%	15%	−4%	−18%	−18%
Non-white population (%, 2013)	66%	67%	60%	65%	48%	41%	45%	41%
Change in non-white population (%, 2000–2013)	22%	14%	23%	3%	33%	21%	35%	31%
Adults (25+) with college degree (%, 2013)	27%	31%	36%	44%	51%	63%	54%	75%
Change in college-educated adult population (%, 2000–2013)	18%	26%	27%	54%	16%	17%	24%	15%
Renter households (%, 2013)	49%	60%	57%	61%	25%	47%	29%	11%
Change in renter households (%, 2000–2013)	21%	9%	6%	−1%	25%	1%	-2%	35%

Note: 11 census tracts designed as college town are not shown in this table.

**Table 4:** The comparison of the three gentrification measures in the San Francisco Bay Area: Urban Displacement Project, Landis, and Freeman, 2000–2013.

		Landis			Freeman		
		Declining (*N* = 56)	Stable (*N* = 1442)	Gentrifying (*N* = 82)	Not Gentrifying (*N* = 92)	Gentrifying (*N* = 97)	Excluded (*N* = 1391)
Low Income	Not losing low-income households or very early stages of displacement (*N* = 329)	55.4%	20.6%	3.7%	21.7%	5.15%	21.8%
	At risk of gentrification or displacement (*N* = 290)	5.4%	20%	1.2%	53.3%	44.3%	14.2%
	Undergoing displacement (*N* = 63)	0.0%	4.2%	3.7%	9.8%	15.5%	2.8%
	Advanced gentrification (*N* = 149)	5.4%	7.8%	42.0%	4.4%	33.0%	8.1%
Medium/High Income	Not losing low-income households or very early stages of displacement (*N* = 476)	30.4%	31.1%	17.3%	4.4%	0.0%	34.0%
	At risk of displacement (*N* = 132)	1.8%	8.0%	21.0%	0.0%	1.0%	9.4%
	Undergoing displacement (*N* = 102)	1.8%	6.4%	11.1%	0.0%	0.0%	7.3%
	Advanced exclusion (*N* = 28)	0.0%	1.9%	0.0%	0.0%	0.0%	2.1%
College-town	N/A (*N* = 11)	0.0%	0.0%	0.0%	6.5%	1.0%	1.0%

## Supplementary Materials

The following are available online at https://www.mdpi.com/1660-4601/16/12/2246/s1, Table S1: Details of Gentrification Measures: Data Sources and Variables.
