# Supplementary material for: Gentrification and Displacement in the San Francisco Bay Area: A Comparison of Measurement Approaches
Source: Int J Environ Res Public Health. 2019 Jun 25;16(12):2246. doi: 10.3390/ijerph16122246 (PMC6616964; doi:10.3390/ijerph16122246)
Supplement: Supplementary file 1 [file ijerph-16-02246-s001.pdf]

Supplementary Table S1: Details of Gentrification Measures: Data Sources and Variables.

Method	Variables	Data Source
Freeman	Median household income<br>% housing stock built in the prior twenty years<br>% urban census tract<br>Median home value<br>% those at least 25 years old possessing a bachelor's degree or higher	U.S. Census or<br>American Community Survey
Landis	Median household income	U.S. Census or<br>American Community Survey
UDP	Population<br>% Housing units in pre-1950 buildings<br>% change in median home sale price<br>% change in rent<br>New market-rate units<br># change in low-income households<br>Change in % low-income in-migration<br>% population growth<br>% low-income households<br>% adults (age 25+) with college degree<br>% renter households<br>% non-white population<br>% change in college-educated adult population<br>Median household income<br>% change in median household income<br>% low-income in-migration	U.S. Census or<br>American Community Survey
	Change in low income households w/ low rent burden	American Community Survey;<br>California Housing Partnership Corporation
	Employment density: jobs per square mile	National Establishment Time-Series
	% change in median home sale price	Dataquick

Freeman: Measure developed by Lance Freeman and classifies areas as gentrifying, non-gentrifying, and excluded; Landis: Landis 3-D methodology, developed by James David Landis, classifies area as stable, declining, and gentrifying; UDP: Urban Displacement Project Regional Early Warning System, which classifies area into eight typologies of gentrification and displacement.
